# Antimicrobial potential of actinomycetes isolated from the unexplored hot Merzouga desert and their taxonomic diversity

**DOI:** 10.1242/bio.035410

**Published:** 2018-08-20

**Authors:** Lahcen Ouchari, Amal Boukeskasse, Brahim Bouizgarne, Yedir Ouhdouch

**Affiliations:** 1 Moroccan-Coordinated Collections of Microorganisms (CCMM), National Center for Scientific and Technical Research (CNRST), Rabat 10170, Morocco; 2 Laboratory of Biology and Biotechnology of Microorganisms, Semlalia Faculty of Sciences, Cadi Ayyad University, Marrakech 10170, Morocco; 3 Laboratory, Plant Biotechnology, Plant Phytochemistry and Microbiology Soil Plants, Faculty of Sciences, Ibn Zohr University, Agadir 10170, Morocco

**Keywords:** Actinobacteria, Merzouga sand dunes, Antimicrobial activities, Repetitive element sequence based polymerase chain reaction (rep-PCR), Taxonomic diversity

## Abstract

The absence of new antibiotics is guiding more and more researchers to specific ecosystems. One hundred and sixty-three *Actinobacteria* isolates were isolated from Merzouga sand and screened for their antibacterial and antifungal activities. To test the antimicrobial effect of isolates, four microorganisms known as human potential pathogens were used. The electrophoretic profiles of isolates obtained by repetitive element PCR fingerprinting (rep-PCR) were compared by clustering. Results showed that among the tested isolates, 59% were active against one or more in testing Gram-positive, Gram-negative and the yeast *Candida albicans*. The importance of culture media for the activity expression was revealed. Comparative analysis of antimicrobial activity divided isolates into 15 groups. The comparison of the average diameters of inhibition zones using Minitab V.17 allowed subdivision of the 15 groups into 20 subgroups. Dendrograms derived from the BOXA1R-PCR fingerprints showed that 36 isolates were grouped in 16 clusters, containing from two to four isolates while 127 isolates were not grouped. The tested antimicrobial activities showed a high biological diversity with important inhibition of pathogens tested. The rep-PCR revealed a high taxonomic diversity of isolates. The combination of antimicrobial activity and rep-PCR results revealed the diverse pattern of Merzouga sand dune *Actinobacteria*.

## Abstract (precis)

**Summary:** Merzouga sand dunes are among Morocco’s extreme environments. This unexplored ecosystem represents a habitat for the isolation of a high diversity of actinomycetes with antimicrobial potential and biotechnological applications.

## INTRODUCTION

Scientists all over the world are endeavoring continuously to search for new antibiotic compounds in order to tackle the serious consequences and dynamic nature of antibiotic resistance. The need for novel bioactive compound discovery is great (Mohammadipanah and Wink, 2016; Vela Gurovic and Olivera, 2017). The total number of bioactive metabolites produced by microorganisms is around 23,000, out of which 10,000 (45% of all bioactive metabolites) are produced by *Actinobacteria* alone. Among this group of bacteria, 7600 (76%) compounds are reported from a single genus: *Streptomyces* (Bérdy, 2012). *Actinobacteria* are prokaryotes, having high guanine and cytosine (GC) content in their DNA, with highly variable metabolic possibilities. The metabolic diversity of the *Actinobacteria* class is due to their extremely large genome, which has hundreds of transcription factors that control gene expression, allowing them to respond to specific needs (Shantikumar et al., 2006). The class *Actinobacteria* comprises five subclasses, 10 orders, 56 families and 286 genera (Ait Barka et al., 2016). They are widely distributed in soils, especially in dry, slightly acidic soils rich in organic matter and represent a high proportion of the soil microbial biomass (Saadoun et al., 2015). *Actinobacteria* are important microorganisms that produce various useful enzymes and secondary metabolites such as immunomodulators, antitumor compounds and antibiotics (Saadoun et al., 2015). It is well known that microbial diversity has not been efficiently explored and the vast majority of prokaryotes (90-99%) present in natural habitats are still to be isolated (Harwani, 2013). Many natural environments are still either unexplored or underexplored and thus can be considered a prolific resource for the isolation of poorly studied microorganisms including rare actinomycetes (Tiwari and Gupta, 2012). Many extremophilic bacteria are recognized to be of industrial interest as potential candidates for future biotechnological applications (Cayol et al., 2015). *Actinobacteria* are known as biofactories of enzymes, with applications in the textile, bio-refineries, food, pulp and paper, agriculture, detergent and pharmaceutical industries (Richa and Vivek, 2018). Arid habitats are among the most plenteous ecosystems with regard to the occurrence of new bacterial species (Mohammadipanah and Wink, 2016). A study of arid, semi-arid and dry Mediterranean soils demonstrated that the diversity of archaea and bacteria are high, regardless of precipitation or vegetation cover (Angel et al., 2010). Analyses of bacterial diversity within the dunes of sandy arid soil in southeast Morocco revealed that *Actinobacteria* (57%) were the most frequent groups (Gommeaux et al., 2010). In addition, *Actinobacteria* are one of the most prolific producers of natural bioactive compounds (Oskay et al., 2004; Tiwari et al., 2015). In this context, Merzouga sand dunes might be a source of rare actinomycetes species. However, the antimicrobial potential and taxonomic diversity of *Actinobacteria* from this habitat has not been investigated.

In this present work, *Actinobacteria* from Merzouga sand dunes were selected and tested for their antimicrobial potential against four microorganisms known as human potential pathogens. The taxonomic diversity of the isolates was evaluated using repetitive element PCR (rep-PCR) fingerprinting in order to show the biodiversity of *Actinobacteria* and the relationship with antimicrobial potential.

## RESULTS AND DISCUSSION

### Isolation of *Actinobacteria*

Classical techniques of plating out suspensions on various solid media that support heterotrophic microorganism growth were used. Among the tested media, Trypton Soy Agar (TSA) revealed the highest total numbers of microbes expressed in colony-forming units (CFU) of bacteria present per gram of sand. *Actinobacteria* were successfully isolated from the sand of Merzouga. Four culture media [TSA, Bennett’s agar, Yeast Malt Extract Agar (YMA) and Sand Extract Agar (SEA)] were used to isolate and reveal the diversity of *Actinobacteria*. The distribution of total bacteria and *Actinobacteria* in the sand dunes is shown in Table 1.

**Table 1.**
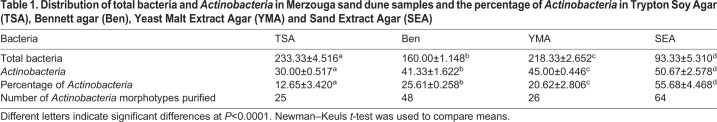
**Distribution of total bacteria and *Actinobacteria* in Merzouga sand dune samples and the percentage of *Actinobacteria* in Trypton Soy Agar (TSA), Bennett agar (Ben), Yeast Malt Extract Agar (YMA) and Sand Extract Agar (SEA)**

The use of different culture media had an important effect on the total number of *Actinobacteria* recovered. On SEA, the sample analyzed contained 90×10^5^ CFU/g and half of the isolates were *Actinobacteria*. Representatives of the *Bacillus* genus were also present as befits inhabitants of hot and dry deserts. Some *Actinobacteria* isolates were colored. Colonies were dark, yellow and greenish yellow. It should be noted however that only heterotrophic and aerobic bacteria can grow on the media tested.

Actinobacterial isolates showed diverse cultural and morphological characteristics: pigment production and color of the substrate and aerial mycelium. One hundred and sixty-three actinobacterial isolates were selected and numbered from one to 163. After successive transfers using the standard microbiological method, isolates grown in TSA media obtained as a pure culture were frozen at −80°C using 20% glycerol for long-term storage. True deserts in which evaporation exceeds rainfall by wide margins have been the main sources of extremophilic microorganisms and airborne dust. Since unexplored and underexplored niche habitats are regarded as biodiversity hotspots (Tiwari et al., 2015), it was speculated that the sand samples of the Merzouga desert could be an excellent target for discovering novel actinobacterial strains producing new bioactive compounds. The total cultivable bacterial count on TSA was 234×10^4^ CFU/g of sand and the total *Actinobacteria* represent ∼13% of the total viable bacteria count on this medium. Similar results have been previously reported by Gommeaux et al. (2010). However, a lower number of bacteria, about 5×10^3^ CFU/g, in the desert of Merzouga were reported by Aanniz et al., (2015).

### Antimicrobial activity of isolated *Actinobacteria*

**Fig. 1.**
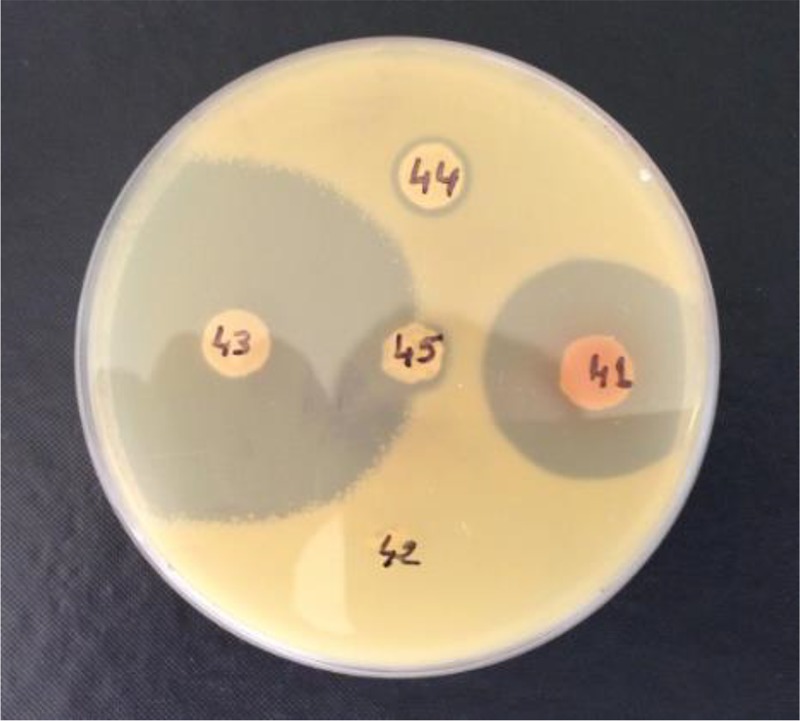
**Antibacterial activity showing clear zone activities on Bennett's agar.**

Actinobacterial isolates were screened for their ability to produce inhibitory bioactive compounds (Fig. 1) against four microorganisms known as human potential pathogens. Two Gram-positive bacteria (*Staphylococcus aureus* and *Listeria monocytogenes*), one Gram-negative bacteria (*Salmonella enterica*) and one yeast (*Candida albicans*) were used to determine the antimicrobial capability of the 163 *Actinobacteria* isolates. Primary screening of antimicrobial activity was performed on TSA using the agar spot method. Among the 163 *Actinobacteria* isolated, 96 (59%) isolates showed antibacterial activities against at least one of the tested microorganisms. Secondary screening of the isolates was examined by the agar spot method on Bennett's agar. Results revealed that 92 (56%) of the isolates were active against at least one of the tested microorganisms. Thirty-four active isolates on TSA became inactive on Bennett's agar; on the other hand, 30 inactive isolates on the same culture medium, TSA, became active on Bennett's agar.

Among the 96 active isolates, 53 are active against either one or both of the Gram-positive pathogens (*S. aureus* and *L. monocytogenes*) and one isolate was only active against the Gram-negative pathogen (*S. enterica*). However, only 30% of isolates showed antifungal activity against *C. albicans*. These results were comparable to those of Ganesan et al. (2017) who isolated 21 actinomycetes from different soil samples and screened them against Gram-positive and Gram-negative bacteria. They found that more than 40% isolates had potential antimicrobial activity against the tested pathogens.

### Frequency and intensity of antibiotic interaction

We examined the frequency of antibiotic interactions between the Merzouga sand dunes’ 163 *Actinobacteria* isolates and the four microorganisms known as human potential pathogens. Frequency was used to summarize the proportion of four tested microorganisms that Merzouga sand isolates could inhibit (presence/absence of inhibition zone). In other words, *Actinobacteria* isolates were grouped based on their antimicrobial activity (active or inactive) against one or more microbial pathogens tested using the statistical program Minitab V.17. Table 2 shows the frequency distribution of phenotypic groups based on the dendrogram generated from qualitative data of antibiotic interaction.

**Table 2.**
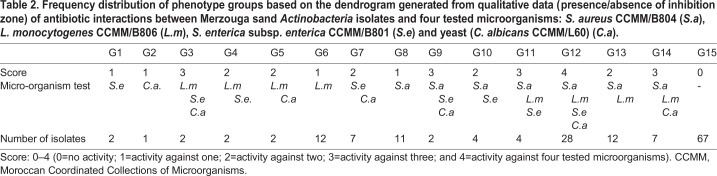
**Frequency distribution of phenotype groups based on the dendrogram generated from qualitative data (presence/absence of inhibition zone) of antibiotic interactions between Merzouga sand *Actinobacteria* isolates and four tested microorganisms: *S. aureus* CCMM/B804 (*S.a*), *L. monocytogenes* CCMM/B806 (*L.m*), *S. enterica* subsp. *enterica* CCMM/B801 (*S.e*) and yeast (*C. albicans* CCMM/L60) (*C.a*).**

Among the 96 actives isolates, 53 are active against one or both Gram-positive pathogens (*S. aureus* and *L. monocytogenes*) and only two isolates against Gram-negative pathogen (*S. enterica*). However, only 30% of isolates showed antifungal activity against *C. albicans* (Table 2).

Twenty-eight active isolates showed a large activity spectrum. For all active isolates, intensity refers to the size of the inhibition zone. The comparison of the average diameters of the inhibition zones of antimicrobial activities revealed the similarity levels among active isolates. Isolates were grouped in several clusters containing from two to 19 isolates and six isolates were not grouped.

According to Table 3, very strong inhibition is shown when the average clear zone is more than 20 mm. About 39 isolates have a very strong inhibitory response against *S. aureus*, 36 against *L. monocytogenes*, 25 against *S*. *enterica* and 34 against *C. albicans*. The wider clear zones were produced by the isolates, against *S. aureus*, *L*. *monocytogenes*, *S*. *enterica* and *C*. *albicans*, were grouped. The clear zones produced against pathogens tested were in the range of 50-60 mm for *S*. *aureus*, 50-60 mm for *L*. *monocytogenes*, 30-40 mm for *S*. *enterica* and 20-30 for *C*. *albicans*. Two isolates were able to inhibit the growth of *S*. *aureus*, *L*. *monocytogenes*, *S*. *enterica* and *C*. *albicans* as wide as 60-76, 40-55, 45-60 and 20-30 mm in diameter, respectively. Other isolates, belonging to different clusters, are able to produce clear zones in the range of 5-50 mm for *S*. *aureus* and *L*. *monocytogenes*, 5-30 mm for *S*. *enterica* and 5-20 mm for *C*. *albicans*.

**Table 3.**
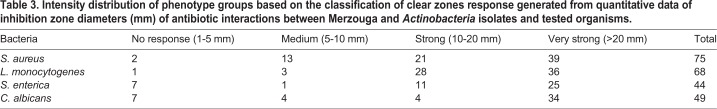
**Intensity distribution of phenotype groups based on the classification of clear zones response generated from quantitative data of inhibition zone diameters (mm) of antibiotic interactions between Merzouga and *Actinobacteria* isolates and tested organisms.**

David and Stout (1971) classified the zone of inhibition (ZOI) in four intensities corresponding to ZOI diameters: >20 mm, very strong; 10-20 mm, strong, 5-10 mm, medium; and <5 mm, no response. The differences in the ability to produce the clear zone were presumably dependent on the secondary metabolites that were produced by test isolates. This assumption was supported by Dharmawan et al. (2009) who stated that the variation of clear zone diameter happens because every isolate produces different types of secondary metabolites. Different types of secondary metabolites have different chemical structures, compounds and chemical concentrations. In this study, the diameters of clear zones produced in four microorganism tests were in the ranges of 20-60, 20-60, 20-40 and 20-30 mm for *S*. *aureus*, *L*. *monocytogenes*, *S*. *enterica* and *C*. *albicans*, respectively. Those ranges of clear zones are classified as having a strong inhibiting response. However, the averages of the clear zones of *S. aureus* and *L*. *monocytogenes* as representatives of Gram-positive are wider than the average of clear zones of *S*. *enterica* as a representative of Gram-negative. It shows that isolates have more ability to inhibit the growth of *S. aureus* and *L. monocytogenes* than to inhibit the growth of *S*. *enterica*. This is because Gram-negative bacteria usually have better protection from other antimicrobial compounds than positive bacteria because both kinds of bacteria have different cell wall components. The cell wall of Gram-positive bacteria contains peptidoglycan, while the cell wall of Gram-negative bacteria contains peptidoglycan and lipopolysaccharide. The statement is supported by Zuhud et al. (2001) and Ajizah and Mirhanuddin (2007), who stated that the cell walls of Gram-positive bacteria contain very thick peptidoglycan for protection. Campbell et al. (1996) added that the cell walls of Gram-negative bacteria, besides peptidoglycan, also contain lipopolysaccharide to protect the bacteria from antibiotics.

### Taxonomical diversity of isolated *Actinobacteria*

**Fig. 2.**
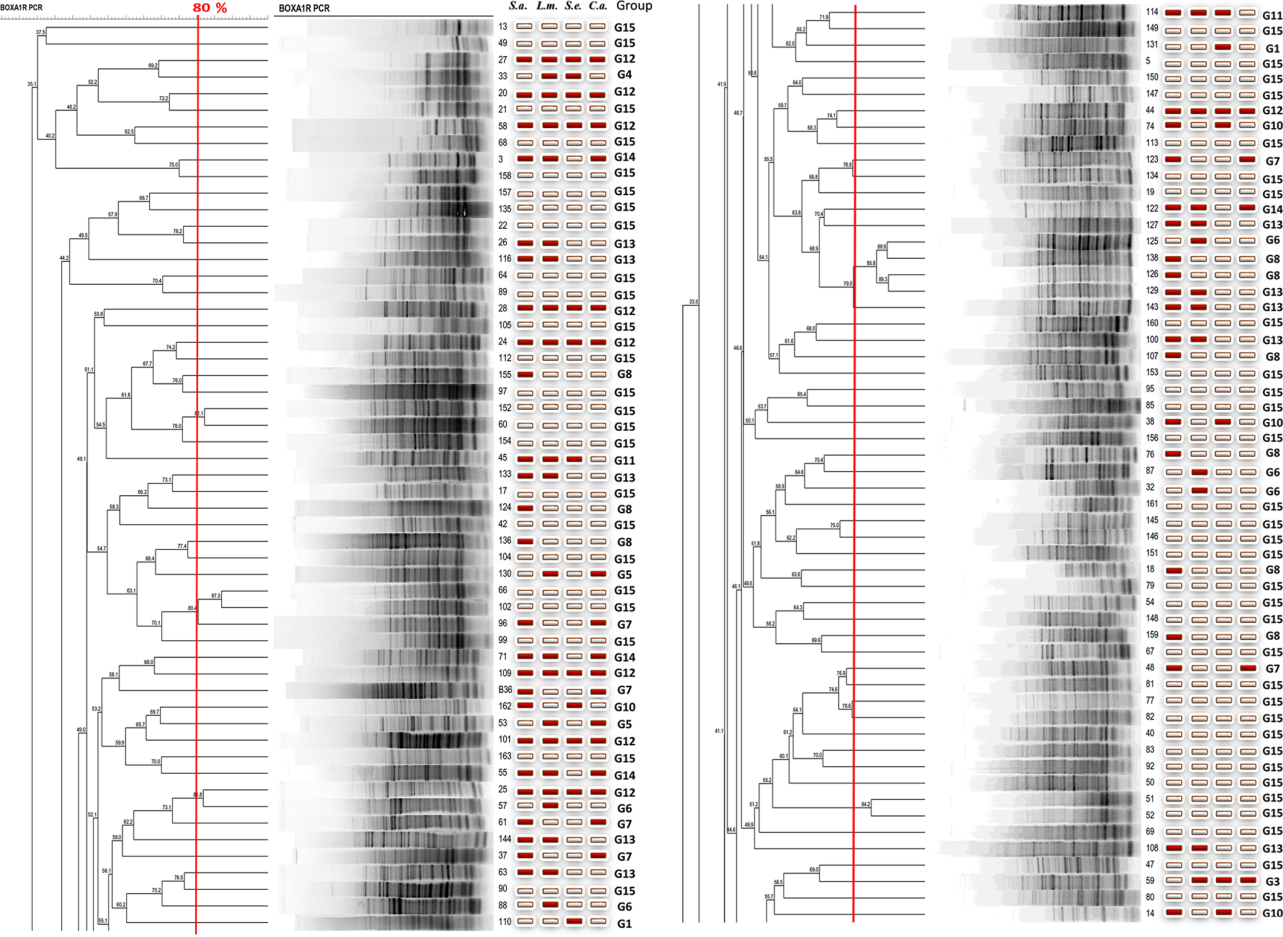

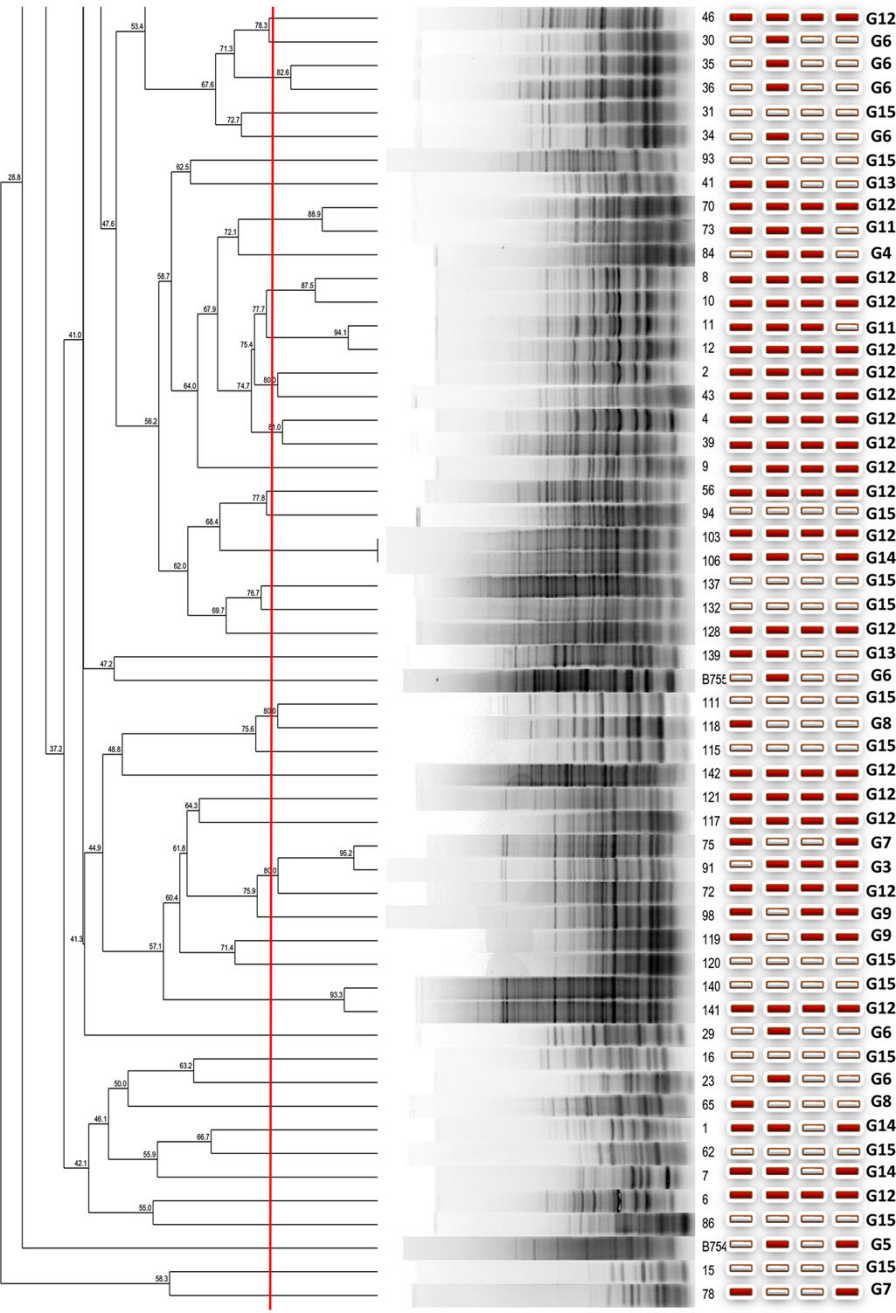
**Dendrogram derived from the BOXA1R-PCR fingerprints of the 163 isolates and their antimicrobial activities (red box, active; white box, inactive).**

The electrophoretic profiles of the Merzouga sand dune isolates and three standard *Actinobacteria* strains obtained by rep-PCR fingerprinting were compared. The dendrogram derived from the BOXA1R-PCR fingerprints (Fig. 2) of the 163 isolates and three standards type strains at significant similarity profiles (80%) showed 16 clusters grouped from two to four isolates. Only 35 isolates were grouped and 128 isolates were not grouped. The profiles were compared to those of three standard type strains of *Streptomyces* genus: (B754) *Streptomyces griseus* subsp. *griseus*, B35 *Streptomyces tinghiriensis* and B755 *Streptomyces rimosus* subsp. *rimosus*). The results showed that Merzouga sand dune *Actinobacteria* isolates were not grouped with these standard strains. These results revealed an interesting taxonomical diversity of isolates suggesting a rich functional population of Merzouga sand dune *Actinobacteria* comparing the antimicrobial activity of the isolates in the same group; isolates belonging to the same cluster showed different antimicrobial activity against the four microorganisms tested.

In this study, the relationship between the activity spectrum and taxonomic diversity was evaluated by comparison of the active isolates showing the same activity spectrum.

In Table 4, frequency distribution of antibiotic phenotypic groups, G1, G2, G6 and G8 grouped active isolates against one microorganism test *S.aureus* (G8), *L*. *monocytogenes* (G6), *S*. *enterica* (G1) and *C*. *albicans* (G2). The main problems for all researchers involved in the screening for new antimicrobial compounds produced by microorganisms is: do the active isolates belong to the same taxonomic group? How do we avoid replication and increase the chemical diversity of produced compounds by the selected isolates during the screening process? In this study, the comparison of the antimicrobial activity of the isolates in the same group to isolates belonging to the same cluster showed different antimicrobial activity against pathogens tested. Although competition, niche partitioning and spatial isolation have been used to describe the ecology and evolution of microorganisms, it is less clear to what extent these principles account for actinobacterial diversity observed in sand dune. Ecological interactions between bacteria are particularly challenging to address due to methodological limitations and uncertainties over how to recognize fundamental units of diversity and link them to the functional traits and evolutionary processes that led to their divergence (Bontemps et al., 2013). In this study, the relationship between the activity spectrum and the taxonomic diversity of the active isolates showing the same score was examined (Table 2); the frequency distribution of antibiotic phenotypic groups, G1, G2, G6 and G8 grouped active isolates against one microorganism [*S. aureus* (G8), *L. monocytogenes* (G6), *S. enterica* (G1) or *C. albicans* (G2)] were tested. This investigation tried to show that the closely related Merzouga sand dune *Actinobacteria* species can be differentiated based on the antimicrobial capability. Using a direct challenge assay to investigate inhibitory interactions with the four human potential pathogens microorganisms, a difference in the onset of inhibition and taxonomic diversity was observed. The majority of antagonistic activity exhibited by the active isolates was linked to antibiotic production. These results support the ecological divergence of the active selected isolates co-occurring and the closely related diversity of Merzouga sand *Actinobacteria* by providing evidence that they have evolved fundamentally different strategies to compete in desert habitats. The BOX-PCR technique, in combination with an extensive database, or 16S rRNA gene sequencing and phylogenetic analysis, may be a valuable identification tools for isolates. As a biotechnological tool, the amplification of bacterial genomic DNA plays an important role in strain improvement in a variety of pharmaceutical and industrial applications, including antibiotic biosynthesis, bioconversion and degradation of toxic compounds (Li et al., 2017). There is a need for the development of new approaches and cultural conditions to recover the actinobacterial strains from arid areas, then advanced or more targeted investigations are required to more fully explore and exploit the abundance, diversity or even the plasticity and function of actinobacterial members in Merzouga desert.

**Table 4.**
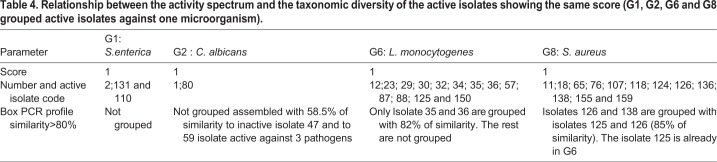
**Relationship between the activity spectrum and the taxonomic diversity of the active isolates showing the same score (G1, G2, G6 and G8 grouped active isolates against one microorganism).**

## CONCLUSION

The *Actinobacteria* class is a very important group of bacteria having a considerable value as prolific producers of antibiotics and other therapeutic compounds. The results of the present study provide further evidence that the Merzouga desert is a rich source of taxonomically diverse, culturable *Actinobacteria* and, potentially, natural products. The 163 actinobacterial isolates were tested against four microbial pathogens and clustered using repetitive DNA fingerprinting. Interestingly, 58% of these isolates showed antibacterial activity and the importance of culture media for activity expression was shown. Based on the antimicrobial activity, isolates showed a high diversity. The dendrogram derived from BOXA1R-PCR profiles, showed a high discriminatory level and revealed an important taxonomical diversity of isolates. The combination between the results of the rep-PCR and the results of the antimicrobial activity revealed the great diversity of *Actinobacteria* isolates in the desert of Merzouga. This biodiversity found could represent a valuable resource for the discovery of biologically active compounds and biotechnological applications.

## MATERIALS AND METHODS

### Site description

**Fig. 3.**
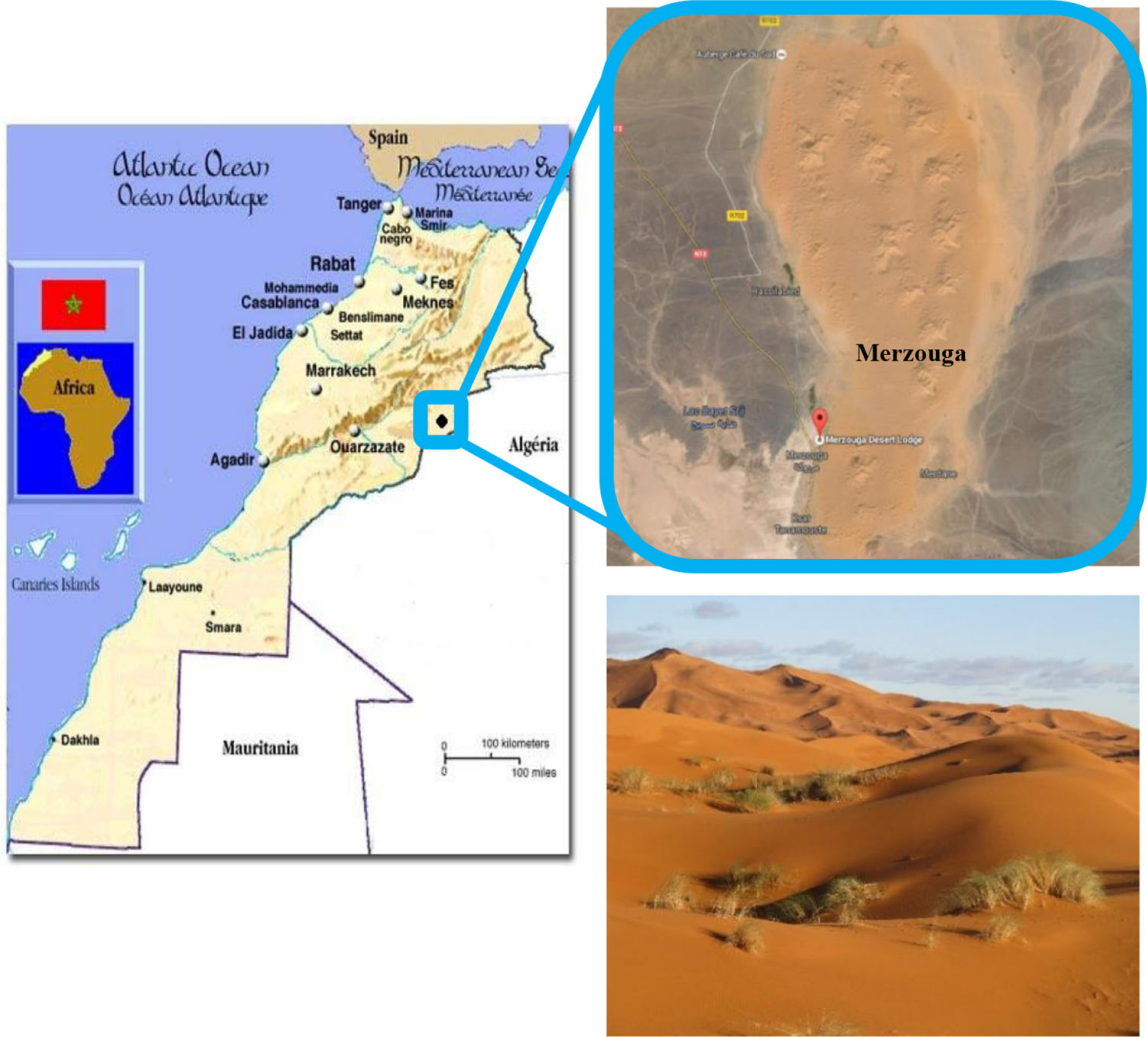
**Geographical overview of Morocco and localization of the Merzouga sand dunes.**

Soil samples were taken from low vegetated sand dunes in Merzouga, southeast of Morocco (31°13′48.1″N/3°58′15.7″W) (Fig. 3). The climate is very dry, with an average of less than eight rainy days per year. The annual rainfall is about 85 mm. From May till August, the region experiences a particularly dry summer. The temperatures are then often higher than 40°C during the day, the nights being cold.

The soil of the Merzouga desert has a sandy texture with a relatively high pH value (pH 9.1) and low humidity (30–50%). As determined from thin section observations under a polarizing microscope (JEOL 6301 scanning electron microscope) the composition of the sand is mostly quartzose (90% in weight). Calcium and iron-carbonates (calcite and siderite) constitute approximately 8% of the sand, derived from the bedrock underlying the dunes. Minorities of iron-oxide grains (1%) are of unknown origin (Gommeaux et al., 2010).

### Sampling and processing

The sand samples were collected from seven different dunes of Merzouga (Fig. 1) (1 kg per dune) using clean, dry and sterile bags along with a sterile spatula. The sample from each dune was taken with an auger (up to 15 cm) after removing approximately 10 cm of the sand surface. The seven collected samples were mixed. After sieve wash using sterile distilled water, sand mixed samples were dried at 45°C for 1 h in a hot air oven and conserved at room temperature before laboratory analysis (BINDER, Tuttlingen, Deutschland). This wash test was used to separate other particles and hydrophilic bacteria from sand. The dried, washed sand was blended in order to improve the isolation of hydrophobic bacteria like *Actinobacteria*, which stuck to the sand wall.

### Isolation and purification of culturable *Actinobacteria*

Samples of blended sand were first mixed, suspended in sterile distilled water (1 g in 10 ml) and shaken on a rotatory shaker (200 revs/min, 30 min) (VELP Scientifica, Usmate, Italy). All treated samples were serially diluted up to 10^−4^ and spread (0.1 ml) over the surface of four culture media: TSA, Bennett's agar, YMA (BIOKAR Diagnostics, Pantin, France) and SEA prepared in the laboratory. The pH was adjusted to 7 and plates were incubated at 28°C for 2 weeks. The number of colonies was determined after 7 days for total bacteria and after 14 days for *Actinobacteria.*

### SEA preparation

Equal volumes of blended sand and distilled water were mixed overnight and filtered after sterilization at 120°C for 15 min. Agar (20 g/l) was added to the filtrate collected and the pH was adjusted to 7.0 before sterilization. To increase the selectivity of this medium the following compounds were added: glycerol (5 g/l) as a carbon source found to be preferable for isolating *Actinobacteria*, nalidixic acid (10 mg/l) (Melford Laboratories, Ipswich, UK) which inhibits the Gram-negative bacteria capable of swarming without affecting the growth of *Actinobacteria* (Bulina et al., 1997) and cycloheximide (40 mg/l) (Melford Laboratories) found to inhibit the growth of fungi. *Actinobacteria* colonies were recognized based on morphological features following directions given by International Streptomyces Project (ISP) (Shirling and Gottlieb, 1966). Most *Actinobacteria* showed a vegetative mycelium and aerial hyphae, others showed only the substrate mycelium. Isolates were purified on TSA and cryopreserved at −80°C with 5% glycerol (Merck KGaA, Darmstadt, Germany).

The statistical analysis of total bacteria and *Actinobacteria* colony formant unit distribution was carried out using ANOVA and the Newman–Keuls test was used to compare the average abundance and the percentage contribution of the *Actinobacteria* to total bacteria in the Merzouga sand dunes. All values are means of three replicate plates.

### Screening for antimicrobial potential

#### Antibacterial and antifungal activity

Actinobacterial isolates were screened for antimicrobial activity using the spot agar method (Rizvi et al., 2014). Two different media were used for growing each isolate, TSA and Bennett's agar. Each plate was spotted with three isolates. Plates were incubated at 30°C for 7-10 days. Two Gram-positive bacteria, *Staphylococcus aureus* CCMM/B804 (*S.a.*) and *Listeria monocytogenes* CCMM/B806 (*L.m.*) and one Gram-negative *Salmonella enterica* subsp *enterica* CCMM/B801) (*S.e.*) was used to test susceptibility to the *Actinobacteria* isolates. Test strains were grown overnight in nutrient broth at 37°C. A suspension 0.5 McFarland of an overnight culture of test bacteria was prepared and inoculated on the plate, close to the *Actinobacteria* spot (Claverías et al., 2015). Plates were incubated at 37°C and after 24 h the zone of inhibition (in mm) around the colonies was measured and registered. For the antifungal activity, the agar overlay method was used. *Actinobacteria* isolates were spot inoculated on TSA plates and incubated for 7 days at 30°C. Colonies were then covered with a 0.6% agar layer of Sabouraud medium previously seeded with a standardized suspension of the testing yeast (*Candida albicans* CCMM/L60) (*C.a.*) and then incubated at 37°C for 24 to 48 h. The zone of inhibition (mm) around the colonies was measured and registered.

#### Characterization of active isolates

In the present investigation, the chemical diversity of the produced molecules by the active isolates was evaluated using antibacterial and antifungal activities. The importance of culture media for the activity expression is studied with two production media, TSA and Bennett's agar. A comparative analysis of antimicrobial active isolates using Minitab V.17 divided the active isolates into groups. The comparison of the average diameters of inhibition zones of all isolates using Minitab V.17 allowed subdividing the previous groups into subgroups.

### Taxonomic diversity

#### Extraction of DNA

DNA extractions were performed using the alkaline lysis method (Bimboim and Doly, 1979). The purified *Actinobacteria* isolates were grown for approximately 5 days on TSA plate. Fresh culture was suspended in 100 μl alkaline lysis solution followed by a heat lysis step for 10 min at 95°C leading to a destruction of cell walls. 180 µl of MiliQ water (Merck KGaA, Darmstadt, Germany) was added and mixed. After centrifugation, the DNA in the supernatant was quantified using NanoDrop 8000 (Thermo Fisher Scientific).

#### Repetitive DNA fingerprinting PCR

Repetitive DNA fingerprinting was performed on all isolates following the method of Versalovic et al. (1994).

The PCR primer derived from the repetitive sequence BOXA1R (5′-CTACGGCAAGGCGACGCTGACG-3′) was used to amplify the DNA samples. Control reactions, without bacterial DNA templates, were included in all the amplifications. The PCR products were separated by electrophoresis on 1.5% agarose gels at 47 V for 18 h at 4°C. Using the Gel Doc XR+ Gel Documentation System (Bio-Rad), stained BOXA1R-PCR gels were visualized under ultraviolet light and gel photographs were stored as TIFF files. These fingerprints were analyzed using BioNumerics software package v7.1 (Applied Maths, Sint Martens Latem, Belgium). Similarity matrices of densitometric curves of the gel tracks were calculated using the Pearson Product Moment Correlation Coefficient (Pearson, 1926) followed by dendrogram construction using an unweighted pair group method with arithmetic mean (UPGMA) algorithm.
